# Author Correction: Cross-kingdom synthetic microbiota supports tomato suppression of Fusarium wilt disease

**DOI:** 10.1038/s41467-023-42557-z

**Published:** 2023-10-24

**Authors:** Xin Zhou, Jinting Wang, Fang Liu, Junmin Liang, Peng Zhao, Clement K. M. Tsui, Lei Cai

**Affiliations:** 1 State Key Laboratory of Mycology, Institute of Microbiology, Chinese Academy of Sciences, Beijing, P. R. China; 2 University of Chinese Academy of Sciences, 100101 Beijing, P. R. China; 3 Faculty of Medicine, University of British Columbia, Vancouver, BC Canada; 4 National Centre for Infectious Diseases, Tan Tock Seng Hospital, Singapore, Singapore; 5 Lee Kong Chian School of Medicine, Nanyang Technological University, Singapore, Singapore; 6 Department of Pathology, Sidra Medicine, Doha, Qatar

**Keywords:** Microbiome, Microbial ecology, Biotic, Metagenomics

Correction to: *Nature Communications* 10.1038/s41467-022-35452-6, published online 22 December 2022

The original version of this article contained errors in Fig. 4. The same image was inadvertently duplicated for panel f and panel g. Panel g has been replaced with a correct image. In addition, in panel i, the data points were misaligned with the trend line.

In addition, there was an error in the first paragraph of the Results section, which incorrectly read ‘The fungal and bacterial compositions (*R* = 0.6317 for bacteria, and *R* = 0.8876 for fungi, *P* < 0.001 for both) of rhizosphere microbial communities clustered into distinct groups that corresponded well to the host biogeography, as determined by analysis of molecular variance (AMOVA) and multivariate analysis of variance (PERMANOVA)’.

The correct version replaces this sentence with ‘The fungal and bacterial compositions (*R* = 0.6317 for bacteria, and *R* = 0.8876 for fungi, *P* = 0.001 for both) of rhizosphere microbial communities clustered into distinct groups that corresponded well to the host biogeography, as determined by analysis of molecular variance (ANOSIM) and multivariate analysis of variance (PERMANOVA)’.

The methods description for ANOSIM was mistakenly omitted from the Supplementary Information. The sentence ‘The differences in the community composition of different groups were also calculated using the analysis of similarities (ANOSIM) (nested “anosim” in vegan R package)^73^’ has now been included.

These errors have been corrected in both the PDF and HTML versions of the article.

### Supplementary information

Updated Supplementary Information

