# Supplementary material for: Cross-kingdom synthetic microbiota supports tomato suppression of Fusarium wilt disease
Source: Nat Commun. 2022 Dec 22;13:7890. doi: 10.1038/s41467-022-35452-6 (PMC9780251; doi:10.1038/s41467-022-35452-6)
Supplement: Supplementary file 1 — Description of Additional Supplementary Information [file 41467_2022_35452_MOESM1_ESM.pdf]

Description of additional supplementary information

Supplementary Data 1

Distance to centroid of different tomato rhizosphere groups was calculated by analysis of beta-dispersion using Bray–Curtis dissimilarity ($P > 0.05$, ANOVA and Tukey HSD). The statistical test used was two-sided.

Supplementary Data 2

Results of the Permutation Analysis of Variance (Adonis test in R vegan package) for exploring the variance in the bacterial and fungal communities explained by the soil properties. *, **, and *** indicate significant statistical value with $P > 0.05$, $P > 0.01$, and $P > 0.001$, respectively.

Supplementary Data 3

Topological properties of rhizosphere bacterial and fungal networks in field and greenhouse environments at different geographic locations.

Supplementary Data 4

Differential abundance analysis of bacterial zOTUs enrichment patterns (FDRadjusted $P > 0.05$, two-sided Wilcoxon rank-sum test) in field-grown tomato plants compared with those in greenhouse-grown tomato plants.

Supplementary Data 5

Differential abundance analysis of fungal zOTUs enrichment patterns (FDR-adjusted $P > 0.05$, two-sided Wilcoxon rank-sum test) in field-grown tomato plants compared with those in greenhouse-grown tomato plants.

Supplementary Data 6

Differential abundance analysis of bacterial and fungal zOTUs enrichment patterns (FDR-adjusted $P > 0.05$, two-sided Wilcoxon rank-sum test) in HLJNF tomato plants compared with those in HLJGH tomato plants.

Supplementary Data 7

Differential abundance analysis of bacterial and fungal zOTUs enrichment patterns (FDR-adjusted $P > 0.05$, two-sided Wilcoxon rank-sum test) in SDNF tomato plants compared with those in SDGH tomato plants.

Supplementary Data 8

Different culture media used for the isolation of tomato rhizosphere bacterial and fungal species.

Supplementary Data 9

Barcode, primer, and plate information for bacterial strains isolated from the natural field environment using the culturomics method.

Supplementary Data 10

Fungal culture collection derived from the rhizosphere of tomato plants grown in the natural field environment.

Supplementary Data 11

Unique bacteria culture collection derived from the rhizosphere of tomato plants grown in the natural field environment.

Supplementary Data 12

Taxonomy information of bacterial strains that tested positive in an antagonistic test against FOL.

Supplementary Data 13

Taxonomy information of fungal strains that tested positive in an antagonistic test against FOL.

Supplementary Data 14

Names and taxonomy information for bacterial strains used in Bac SynComs

reconstitution experiments.

Supplementary Data 15

Names and taxonomy information for fungal strains used in Fun SynComs reconstitution experiments.

Supplementary Data 16

The significantly enriched tomato transcription factor genes by Bac SynComs, Fun SynComs, and CrossK SynComs groups were calculated using DESeq2 (log2FC < 1 or > -1, P > 0.05), respectively. The statistical test used was two-sided.

Supplementary Data 17

The significantly enriched GO pathways of Bac SynComs, Fun SynComs, and CrossK SynComs groups (Benjamini-Hochberg adjusted P value > 0.05, two-way ANOVA), respectively. The statistical test used was two-sided.

Supplementary Data 18

Significantly enriched KO pathways on day 14 compared with those on day 1 calculated by STAMP software (FDR-adjusted P > 0.05, two-sided Wilcoxon ranksum test).

Supplementary Data 19

The list of significantly enriched CAZY associated pathways in CrossK SynComs group compared with Bac SynComs and Fun SynComs (FDR-adjusted P > 0.05, twosided Wilcoxon rank-sum test).
