# Supplementary material for: Cross-kingdom synthetic microbiota supports tomato suppression of Fusarium wilt disease
Source: Nat Commun. 2022 Dec 22;13:7890. doi: 10.1038/s41467-022-35452-6 (PMC9780251; doi:10.1038/s41467-022-35452-6)
Supplement: Supplementary file 2 — Supplementary Information [file 41467_2022_35452_MOESM2_ESM.pdf]

Supplementary information

Cross-kingdom synthetic microbiota supports tomato suppression of Fusarium wilt disease

Xin Zhou[1,2], Jinting Wang[1,2], Fang Liu[1], Junmin Liang[1], Peng Zhao[1], Clement K.M. Tsui[3,4,5], Lei Cai[1,2]*

*Correspondence to: cail@im.ac.cn

Supplementary Methods

Microbial diversity, taxonomic and statistical analysis

The fungal ITS1 region and bacterial 16S rRNA gene sequences were separately processed using USEARCH11 software and VSEARCH software, respectively[70,71]. In brief, the acquired 16S rRNA and ITS1 sequences were quality-filtered and merged into a single sequence using USEARCH11 pipelines[70]. Bacterial and fungal chimeric sequences were detected and removed using the UCHIME algorithm in USEARCH11 against the Ribosomal Database Project (RDP) Gold database UNITE CHIME reference dataset[72], respectively. Then, all nonchimeric sequences were sorted by abundance, dereplicated, and clustered to zOTUs using 'unoise3' algorithm with default parameters in USEARCH11[71]. Bacterial and fungal zOTUs with reads fewer than 8 were removed, and their representative sequences were annotated to taxonomic categories using the 'sintax' and RDP Naive Bayesian Classifier algorithms within the SILVA 138 database and UNITE database at a confidence threshold of 0.8, respectively[69,70]. All fungal and bacterial ZOTUs assigned only to a kingdom were removed to avoid an overestimation of microbial diversity. The rarefaction curves of bacterial and fungal samples were calculated with the 'rarecurve' function in vegan, respectively[73]. The rarefaction curves of fungal communities and bacterial communities by the observed zOTUs showed that most samples nearly approached an asymptote, indicating the sufficient of sequencing depth. Cumulative sum scaling (CSS) was used as a normalization algorithm for diversity analyses of bacterial and fungal communities, to allow the comparison on an equal basis. The alpha diversities were calculated based on the species richness index to estimate the bacterial and fungal species richness[74]. Bray-Curtis dissimilarity matrices between samples were calculated, visualized, and plotted using principal coordinate analysis (PCoA) or Principal Component Analysis to present

the dissimilarities among different samples. Permutational multivariate analysis of variance (PERMANOVA) statistical tests followed by Tukey Honest Significant Difference (HSD) method were implemented to determine the effects of different factors on the community dissimilarity using beta distance matrices (nested "adonis" in vegan R package)[73]. The differences in the community composition of different groups were also calculated using the analysis of similarities (ANOSIM) (nested "anosim" in vegan R package)[73]. The Kruskal–Wallis test and Tukey post hoc test when appropriate ($P < 0.05$) were used for the comparison of field and greenhouse groups. Additionally, differential abundance analysis between NF and GH was calculated using the negative binomial generalized linear model in R package edgeR[30]. We used the trimmed mean of M-values (TMM) normalization method and a False Discovery Rate (FDR) corrected value of $P < 0.05$. Random Forest machine learning classification analysis was employed to acquire the best discriminant performance of biomarkers across NF and GH tomato plants using the randomForest package v.4.7–1[80]. The bacterial and fungal communities of tomato plants at different taxonomic levels (phylum, class, order, family, and genus) were calculated separately to obtain the best discriminating biomarkers with the highest classification accuracy[25]. For the prediction of different taxonomic levels, the randomForest (ntree = 1000, importance = TRUE, proximity = TRUE) function was employed to generate the classification model for NF and GH tomato plants. Cross-validation was performed using rfcv function (ten repeats) for selecting appropriate biomarkers, and the varImpPlot function was used to show the importance of biomarkers in the classification[80].

Co-occurrence network analysis and definition of keystone taxa of NF tomato

The co-occurrence network analysis was performed using the bacterial and fungal zOTUs with relative abundance greater than 0.1%. The non-parametric Spearman correlation analysis were used to reconstruct the co-occurrence patterns and calculate the topological network properties[75]. The co-occurrence networks were regarded as robust if the Spearman's correlation coefficient (ρ) > 0.70 and the significant P value < 0.05. The P values were adjusted with the minimize false positive signals using Benjamini–Hochberg procedure[76]. The important network topological parameters including the number of edges, average path length, average degrees, number of vertices were calculated and visualized to compare the microbial networks differences of GH and NF tomato plants. The ecologically important keystone microbes frequently co-occur with other microbes in microbial networks and potentially play important roles in the microbial community[77]. We reveal the keystone microbes of NF tomato plants based on the differences in co-occurrence network interactions between GH- and NF-tomato microbiomes by employing the online platform NetShift (https://web.rniapps.net/netshift)[33].

The NetShift analysis could find the significant overall change in microbial communities and associations of each node (taxon) in healthy and diseased groups. The keystone taxa could be determined based on the node size and NESH score. NESH score is a Neighbor Shift score that could quantify directional changes in the individual interactions, and each node represents a taxon. The size of each node represents their NESH score, and the red color node indicates its betweenness increases from healthy group to disease group. Thus, the big and red nodes indicate the potential keystone taxa[33].

Phylogenetic tree of most abundant fungal and bacterial zOTUs

The most abundant fungal and bacterial zOTUs (relative abundance ＞ 0.1%) were chosen, with 167 fungal and 266 bacterial zOTUs and associated representative sequences were used for the construction of maximum likelihood (ML) trees. The IQ-Tree software was used for the ML tree construction with the Best-fit model TIM3e+I+G4, following parameters 5000 Ultrafast bootstrap and 1000 SH-like approximate likelihood ratio test[78]. The tree files were uploaded to the iTOL (http://itol.embl.de)[79] online and the phylogenetic trees were edited, and annotated with the heatmaps of the relative abundance of zOTUs in four different locations in the phylogenetic tree. The isolated bacterial and fungal strains which were classified into the same genera presented in the phylogenetic trees were added to the outer rings as pink dots, respectively.

Metagenome quality filtering and annotation pipelines

Twenty-four different tomato samples of different SynComs were chosen for metagenomic sequencing using the Illumina NovaSeq 6000 instrument (Majorbio Bio-pharm Technology, Shanghai, China). The entire data processing pipeline and scripts were made available at GitHub (https://github.com/XinJason/Cross-kingdom-synthetic-microbiota). The low-quality raw data were stripped, trimmed (length<50 bp or with a quality value <20 or having N bases) and removed by Trimmomatic[81]. To remove host (*Solanum lycopersicum*) sequences, Bowtie2 v2.4.1[82] was used to build a host genome database. All reads aligned to the host genome and their mated reads were comprehensively removed using Bowtie2[82]. In total, 0.29% to 4.44% of the clean reads were removed. After removal of nonmicrobial sequences, the remaining sequences were taxonomically assigned using MetaPhlAn2 with the "very sensitive" global alignment option. The relative abundance of gene ortholog groups and functional pathways were generated using HUMAnN2 v2.8.1 against the utility_mapping, chocophlan, and uniref90 databases, respectively[83]. The HUMAnN2 output tables were merged across all sample using humann2_join_tables scripts, and were normalized to counts per million (CPM) before

downstream application using humann2_renorm_table script. The comparison of each of the resulting pathways was conducted using the normalized abundance tables using one-way ANOVA test and Tukey HSD. The filtered reads were assembled to different contigs using MEGAHIT v1.2.9[84]; the gene catalogs were predicted and clustered over contigs by using Prokka and CD-HIT (v4.8.1) to generate a non-redundant gene catalog, respectively[85]. The functional annotations were performed by eggnog-mapper v0.13.1[86] using DIAMOND software[87] and eggNOG databases[88]. The functional annotation results were reorganized into KEGG orthologs (KOs) profiles[89], clusters of orthologous group of proteins categories (COG) [90], and CAZymes[91]. The antibiotic resistance genes were reorganized and annotated using ResFams[92]. The KO abundance within each sample were normalized by the median universal single-copy gene abundance. The STAMP[93] and Linear discriminant analysis (LDA) effect size (LEfSe) software[94] were implemented to analyze statistically significant differential abundance of functional genes or pathways corresponding to different SynCom groups.

RNA seq of tomato plants

For the transcriptional analysis, the tomato leaves treated with different SynComs and *FOL*, were harvested separately in three biological replicates at 7 dpt. The total RNA extraction and reverse transcription methods followed the procedure described above. The sequencing libraries were constructed using the TruSeq Stranded Total RNA kit (Illumina, RS–122–2402) and sequenced using the Illumina NovaSeq 6000 instrument (Paired-end 2 × 150 bp) (Majorbio Bio-pharm Technology, Shanghai, China). Clean reads were obtained by filtering low-quality reads as well as reads containing poly-N sequences or adaptor sequences from raw data. The percentages of Q20 and Q30 reads was calculated from clean sequences using MultiQC v0.4[95], and the remaining high-quality sequences were used for downstream analyses. The clean reads were mapped to the reference genome of tomato (*Solanum lycopersicum*, genome ID: GCF_000188115.3_SL2.50) using HISAT2 v2.2.0[96], and the mapped sequences were aligned and sorted using SAMtools v1.3.1[97]. The gene expression levels of each sample were estimated as FPKM (fragments per kilobase of transcript per million fragments) mapped by the Salmon v0.8.2[98]. Differential expressions of transcripts in different tomato samples were calculated as log2 fold-change (LFC) using the "DESeq2" package[99]. Differential expressions between different treatments were tested against the null hypothesis LFC < 2 with Benjamini and Hochberg adjusted $P < 0.05$, respectively. To compare the gene ontology processes of tomato plants involved in different SynComs treatments, GO terms from using DESeq2 results of each of the groups were extracted with $P > 0.05$ and $-1 \leq \log2$ fold-change ≤ 1. Based on genes significantly (FDR > 0.05) up-regulated in different synthetic microbiota treatments, we

estimated GO term enrichment for Biological Processes and Molecular Functions using GENEONTOLOGY online software (http://geneontology.org/). The enriched GO terms were visualized using the ImageGP platform[100].

Reference

70. Rognes, T., Flouri, T., Nichols, B., Quince, C. & Mahé, F. VSEARCH: a versatile open source tool for metagenomics. *PeerJ* **4**, e2584 (2016).

71. Edgar, R. Taxonomy annotation and guide tree errors in 16S rRNA databases. *PeerJ* **6**, e5030 (2018).

72. Nilsson, R. H. et al. The UNITE database for molecular identification of fungi: handling dark taxa and parallel taxonomic classifications. *Nucleic Acids Res.* **47**, D259–D264 (2019).

73. Oksanen, J. et al. vegan: Community Ecology Package. (2016).

74. Jost, L. Entropy and Diversity. *Oikos* **113**, 363–375 (2006).

75. Gao, M. et al. Disease-induced changes in plant microbiome assembly and functional adaptation. *Microbiome* **9**, 187 (2021).

76. Benjamini, Y. & Hochberg, Y. Controlling the False Discovery Rate: A Practical and Powerful Approach to Multiple Testing. *Journal of the Royal Statistical Society: Series B (Methodological)* **57**, 289–300 (1995).

77. van der Heijden, M. G. A. & Hartmann, M. Networking in the Plant Microbiome. *Plos Biol.* 2016 (10.1371/journal.pbio.1002378).

78. Nguyen, L. T., Schmidt, H. A., von Haeseler, A. & Minh, B. Q. IQ-TREE: a fast and effective stochastic algorithm for estimating maximum-likelihood phylogenies. *Mol. Biol. Evol.* **32**, 268–274 (2015).

79. Letunic, I. & Bork, P. Interactive Tree Of Life (iTOL) v4: recent updates and new developments. *Nucleic Acids Res.* **47**, W256–W259 (2019).

80. Liaw, A. & Wiener, M. Classification and regression by randomForest. *R News* **2**, 18–22 (2002).

81. Bolger, A. M., Lohse, M. & Usadel, B. Trimmomatic: a flexible trimmer for Illumina sequence data. *Bioinformatics* **30**, 2114–2120 (2014).

82. Langmead, B. & Salzberg, S. L. Fast gapped-read alignment with Bowtie 2. *Nat. Methods* **9**, 357–359 (2012).

83. Segata, N. et al. Metagenomic microbial community profiling using unique clade-specific marker genes. *Nat. Methods* **9**, 811–814 (2012).

84. Li, D., Liu, C.-M., Luo, R., Sadakane, K. & Lam, T.-W. MEGAHIT: an ultra-fast single-node solution for large and complex metagenomics assembly via succinct de Bruijn graph. *Bioinformatics* **31**, 1674–1676 (2015).

85. Seemann, T. Prokka: rapid prokaryotic genome annotation. *Bioinformatics* **30**, 2068–2069 (2014).

86. Huerta-Cepas, J. et al. Fast Genome-Wide Functional Annotation through Orthology Assignment by eggNOG-Mapper. *Mol. Biol. Evol.* **34**, 2115–2122 (2017).

87. Buchfink, B., Xie, C. & Huson, D. H. Fast and sensitive protein alignment using DIAMOND. *Nat. Methods* **12**, 59–60 (2015).

88. Huerta-Cepas, J. et al. eggNOG 5.0: a hierarchical, functionally and phylogenetically annotated orthology resource based on 5090 organisms and 2502 viruses. *Nucleic Acids Res.* **47**, D309–D314 (2019).

89. Ogata, H. et al. KEGG: Kyoto Encyclopedia of Genes and Genomes. *Nucleic Acids Res.* **27**, 29–34 (1999).

90. Tatusov, R. L. The COG database: a tool for genome-scale analysis of protein functions and evolution. *Nucleic Acids Res.* **28**, 33–36 (2000).

91. Yin, Y. et al. dbCAN: a web resource for automated carbohydrate-active enzyme annotation. *Nucleic Acids Res.* **40**, W445–W451 (2012).

92. Gibson, M. K., Forsberg, K. J. & Dantas, G. Improved annotation of antibiotic resistance determinants reveals microbial resistomes cluster by ecology. *ISME J.* **9**, 207–216 (2015).

93. Parks, D. H., Tyson, G. W., Hugenholtz, P. & Beiko, R. G. STAMP: statistical analysis of taxonomic and functional profiles. *Bioinformatics* **30**, 3123–3124 (2014).

94. Paulson, J. N., Stine, O. C., Bravo, H. C., & Pop, M. Differential abundance analysis for microbial marker-gene surveys. *Nat. Methods* **10**, 1200–1202 (2013).

95. Ewels, P., Magnusson, M., Lundin, S. & Käller, M. MultiQC: summarize analysis results for multiple tools and samples in a single report. *Bioinformatics* **32**, 3047–3048 (2016).

96. Kim, D., Langmead, B. & Salzberg, S. L. HISAT: a fast spliced aligner with low memory requirements. *Nat. Methods* **12**, 357–360 (2015).

97. Li, H. et al. The Sequence Alignment/Map format and SAMtools. *Bioinformatics* **25**, 2078–2079 (2009).

98. Patro, R., Duggal, G., Love, M. I., Irizarry, R. A. & Kingsford, C. Salmon provides fast and bias-aware quantification of transcript expression. *Nat. Methods* **14**, 417–419 (2017).

99. Love, M. I., Huber, W. & Anders, S. Moderated estimation of fold change and dispersion for RNA-seq data with DESeq2. *Genome Biol.* **15**, 1–21 (2014).

100. Chen, T., Liu, Y. X., & Huang, L. ImageGP: An easy-to-use data visualization web server for scientific researchers. *iMeta* **1**, e5 (2022).

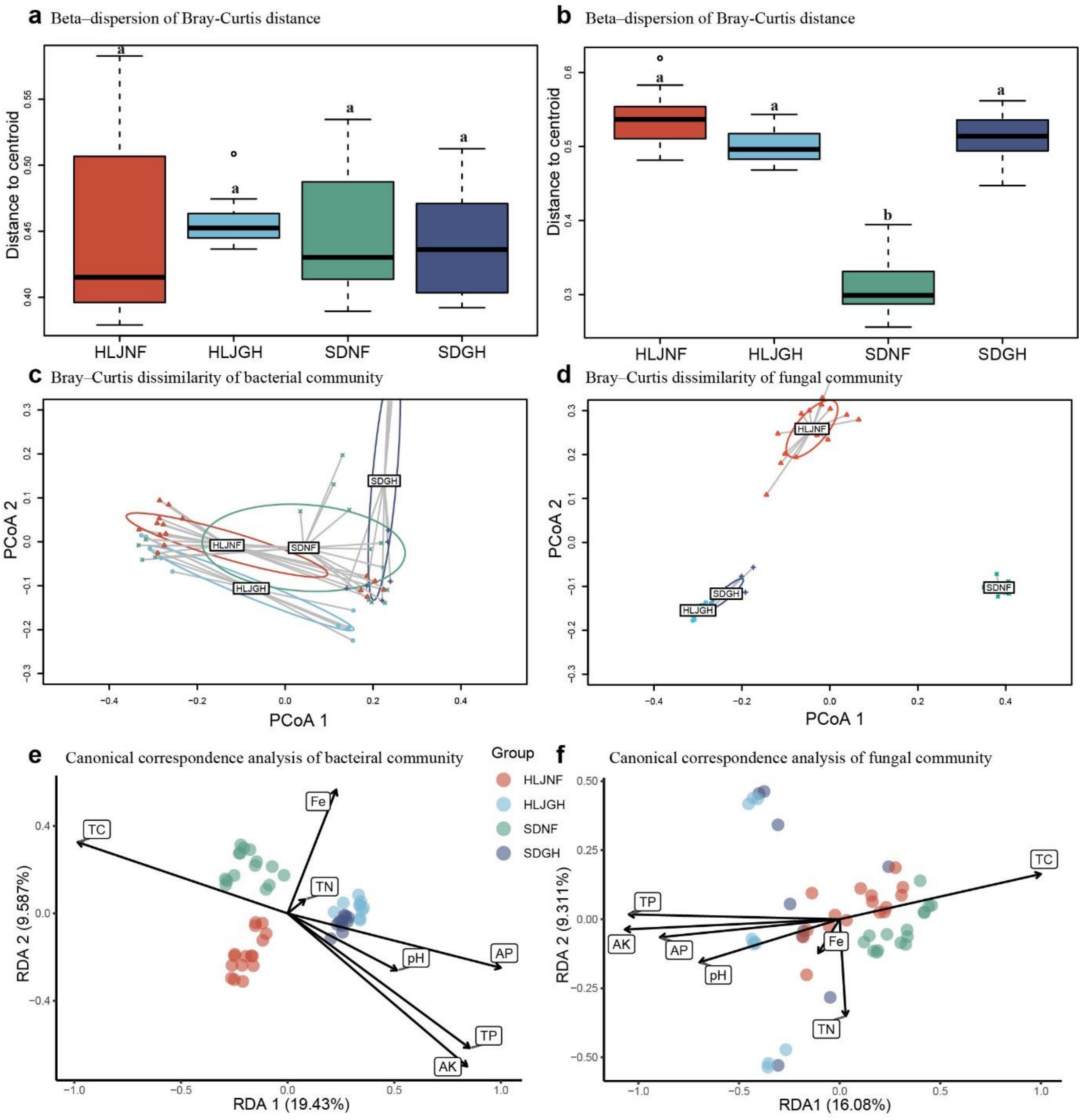

Supplementary Fig. 1 Beta dispersion (distance to group centroid) of bacterial (**a**) and fungal (**b**) communities among different field (HLJNF and SDNF) and greenhouse tomato groups (HLJGH and SDGH) ($P < 0.05$, two-way ANOVA and Tukey HSD). In a-b, the central bars represent median values, tops and bottoms of boxes represent the 75th and 25th percentiles; and upper and lower whiskers extend to data no more than 1.5 times of the interquartile range from the upper edge and lower edge of the box, respectively. Point value beyond this range is plotted as individual points. (**c-d**), Principal coordinate analysis (PCoA) plots of bacterial (**c**) and fungal (**d**) Bray–Curtis dissimilarity distance among different field and greenhouse tomato groups in two provinces (Heilongjiang and Shandong provinces). (**e-f**), The RDA ordination plot of significant soil physicochemical properties associated with bacterial communities (**e**) and fungal communities (**f**) in field and greenhouse tomato groups. The number of samples per

group is as follows: HLJNF (n = 16 biologically independent plants), HLJGH (n = 10 biologically independent plants), SDNF, (n = 15 biologically independent plants), and SDGH (n = 10 biologically independent plants). Vectors show fitted values of soil physicochemical properties significantly correlated within ordination space. The correlations between the soil physicochemical properties and RDA axes are represented by the length and angle of the arrows. HLJNF, the NF rhizosphere of Heilongjiang province (red color); HLJGH, the GH rhizosphere of Heilongjiang province (cyan color); SDNF, the NF rhizosphere of Shandong province (green color); SDGH, the GH rhizosphere of Shandong province (blue color).

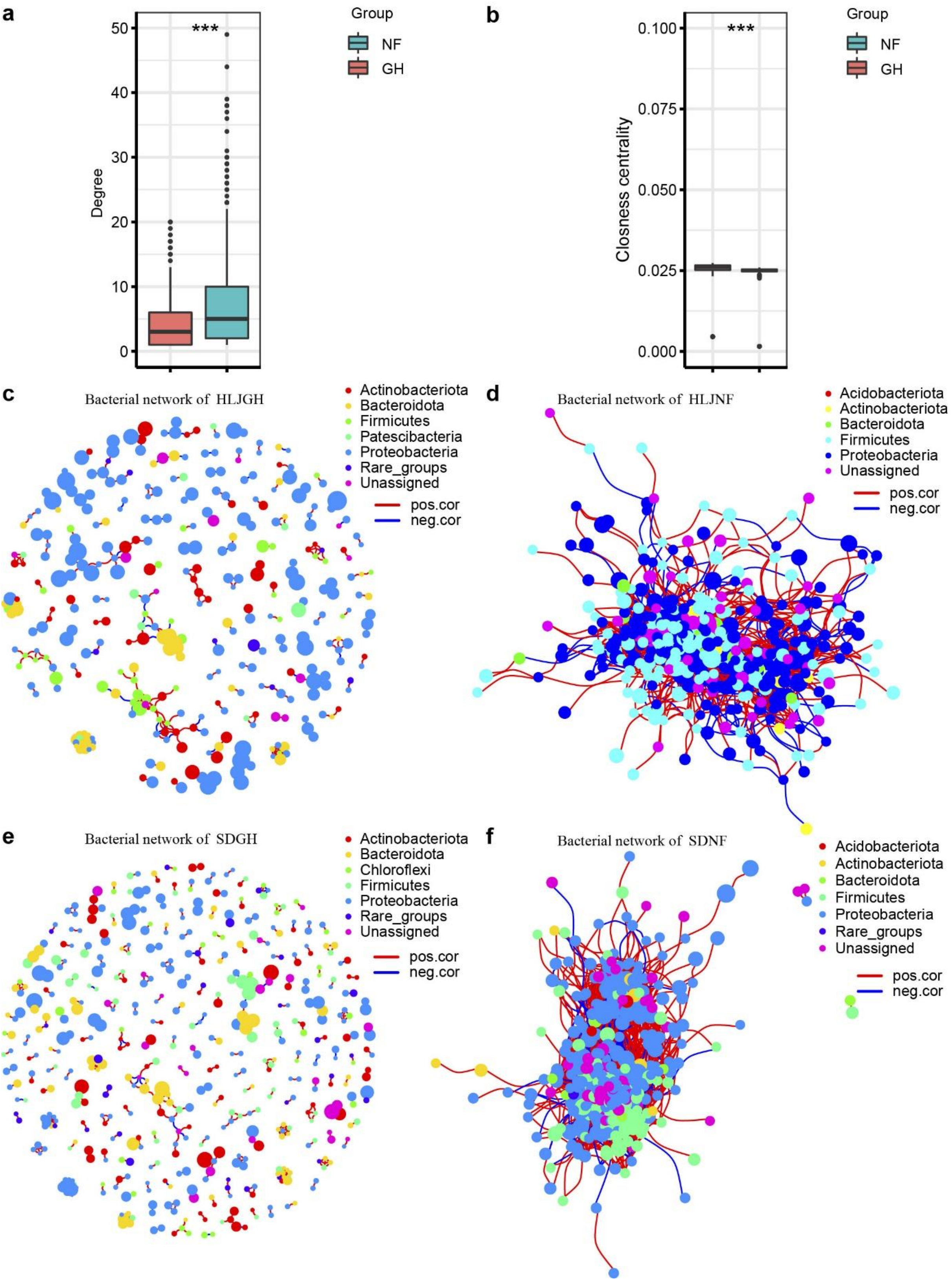

Supplementary Fig. 2 Visualization of the co-occurrence networks of bacteria from tomato groups of field-grown (NF) and greenhouse-grown (GH) tomato plants. Degree (**a**) and closeness centrality (**b**) of bacterial co-occurrence networks were significantly higher than those of GH tomato plants for both bacteria (P < 0.001, Wilcoxon–Wilcox test). NF (n = 31

biologically independent plants), GH (n = 20 biologically independent plants). (**c**) Co-occurrence networks of bacterial communities of HLJGH tomato. (**d**) Co-occurrence networks of bacterial communities of HLJNF tomato. (**e**) Co-occurrence networks of bacterial communities of SDGH tomato. (**f**) Co-occurrence networks of bacterial communities of SDGH tomato. Nodes represent individual zOTUs, with the bacterial phyla indicated by different colors. Links between nodes indicate significant correlations between zOTUs.

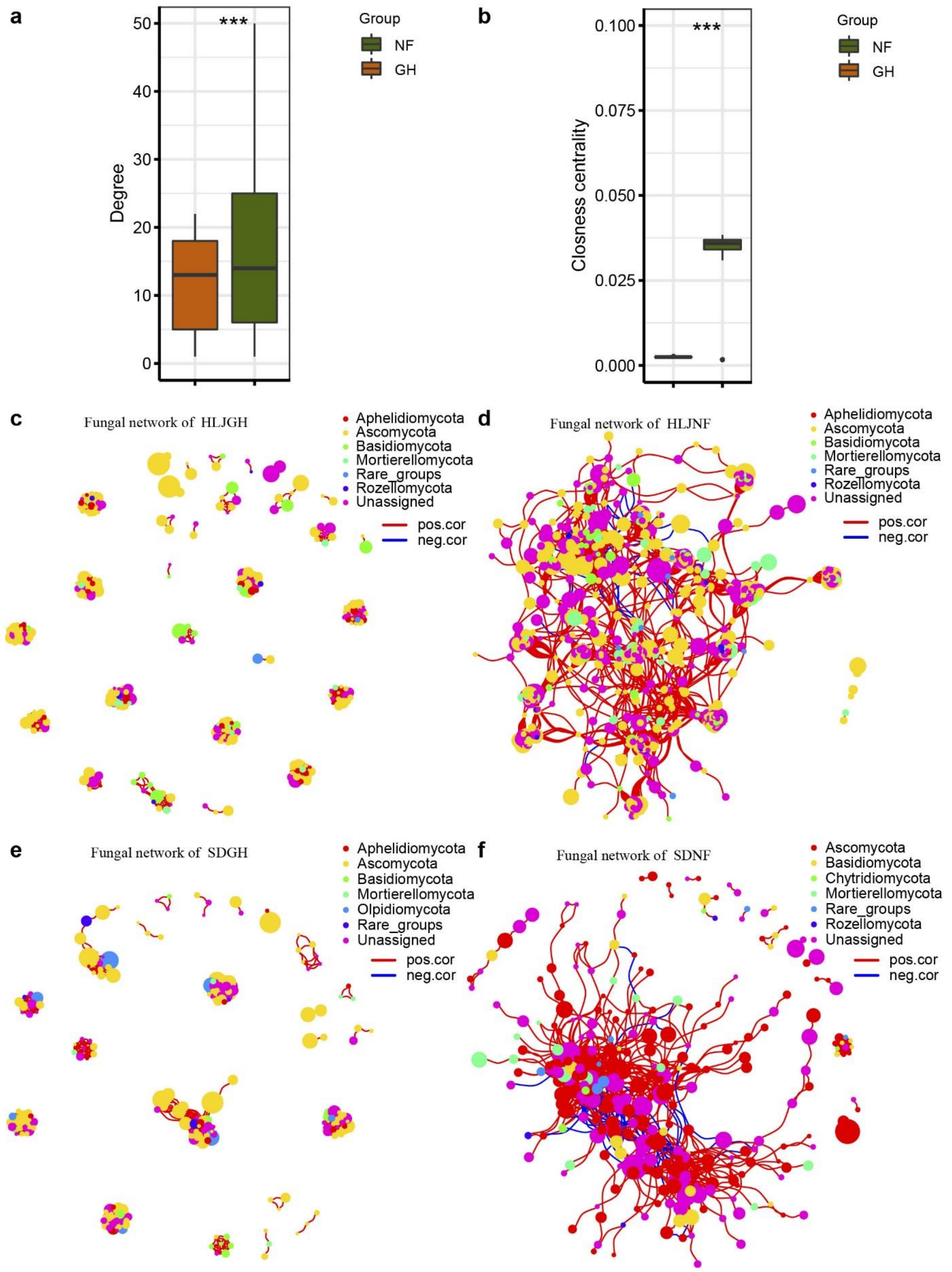

Supplementary Fig. 3 Visualization of the co-occurrence networks of fungi from tomato groups of field-grown (NF) and greenhouse-grown (GH) tomato plants. Degree (**a**) and closeness centrality (**b**) of fungal co-occurrence networks in NF and GH tomato plants were significantly higher than those of GH tomato plants for both bacteria ($P < 0.001$, Wilcoxon–

Wilcox test) (n = 31 biologically independent plants), GH (n = 20 biologically independent plants). (**c**) Co-occurrence networks of fungal communities of HLJGH tomato. (**d**) Co-occurrence networks of fungal communities of HLJNF tomato. (**e**) Co-occurrence networks of fungal communities of SDGH tomato. (**f**) Co-occurrence networks of fungal communities of SDGH tomato. Nodes represent individual zOTUs, with the fungal phyla indicated by different colors. Links between nodes indicate significant correlations between zOTUs.

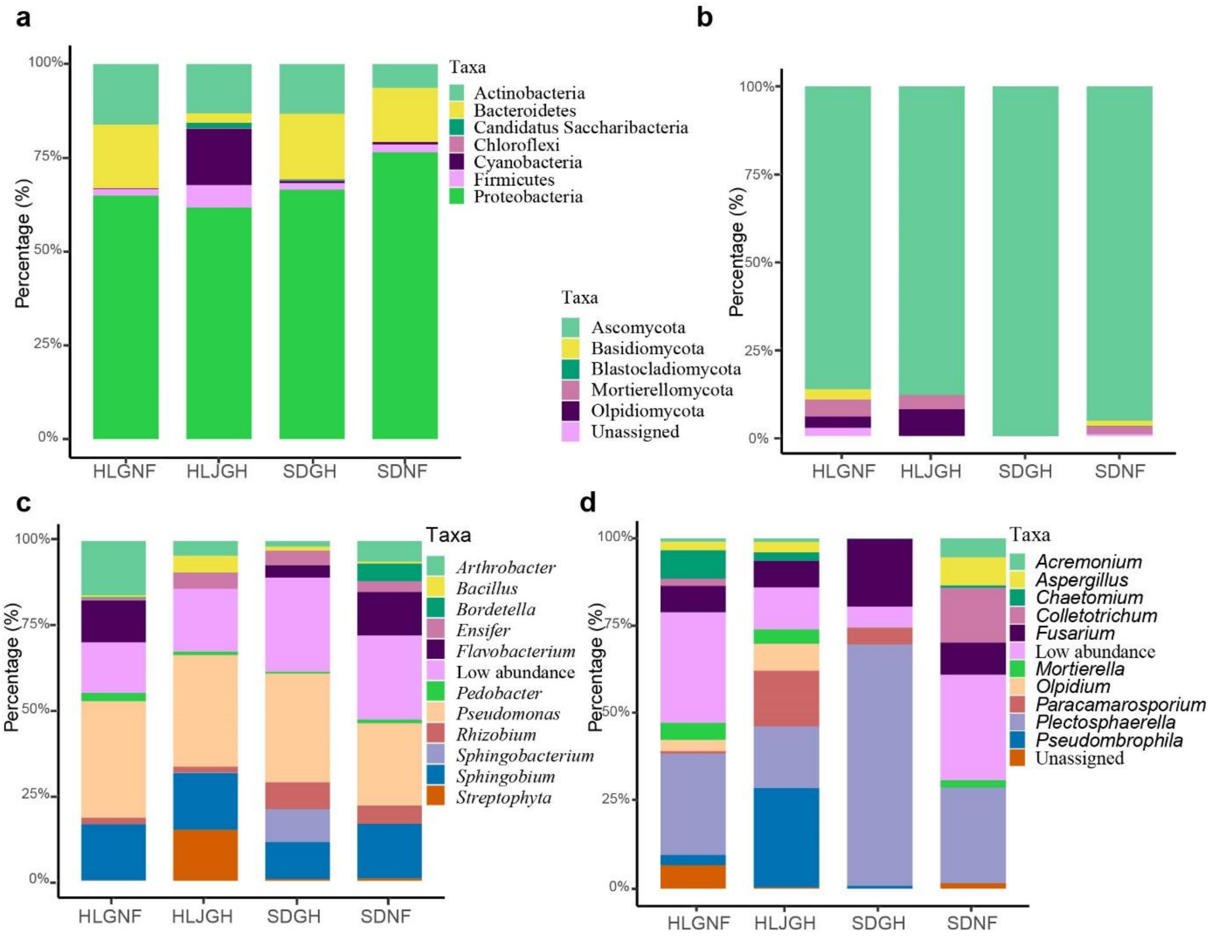

Supplementary Fig. 4 The relative abundance of bacteria and fungi at phylum and genus levels. The relative abundance of dominant bacterial taxa (**a**) and fungal taxa (**b**) in different field and greenhouse groups at phylum level. Relative abundance of dominant bacterial genus (**c**) and fungal genus (**d**) in different field and greenhouse tomato groups. HLJNF, the NF rhizosphere of Heilongjiang province; HLJGH, the GH rhizosphere of Heilongjiang province; SDNF, the NF rhizosphere of Shandong province; SDGH, the GH rhizosphere of Shandong province.

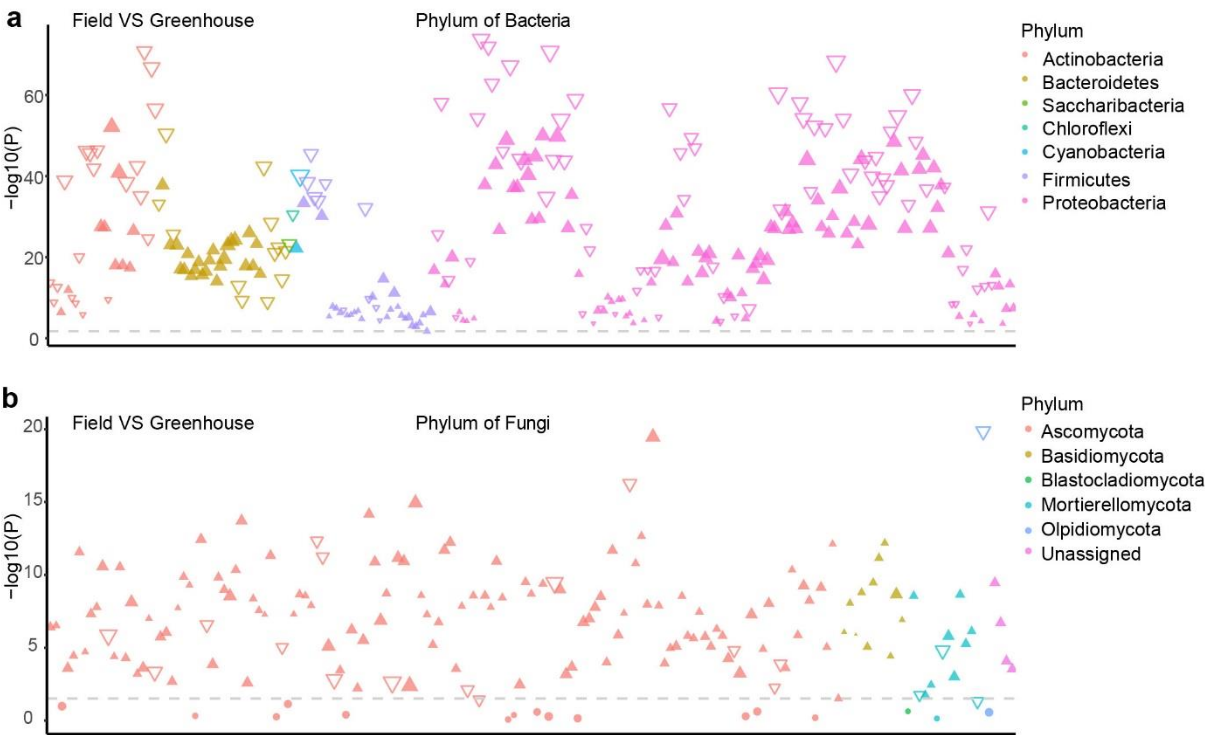

Supplementary Fig. 5 The significantly enriched bacterial and fungal taxa of field and greenhouse environments, revealed by edgeR. Manhattan plots presenting significantly enriched and depleted bacterial taxa (**a**) and fungal taxa (**b**) in NF tomato compared with those in GH tomato groups in both provinces (FDR adjusted $P < 0.05$, two-sided Wilcoxon rank sum test).

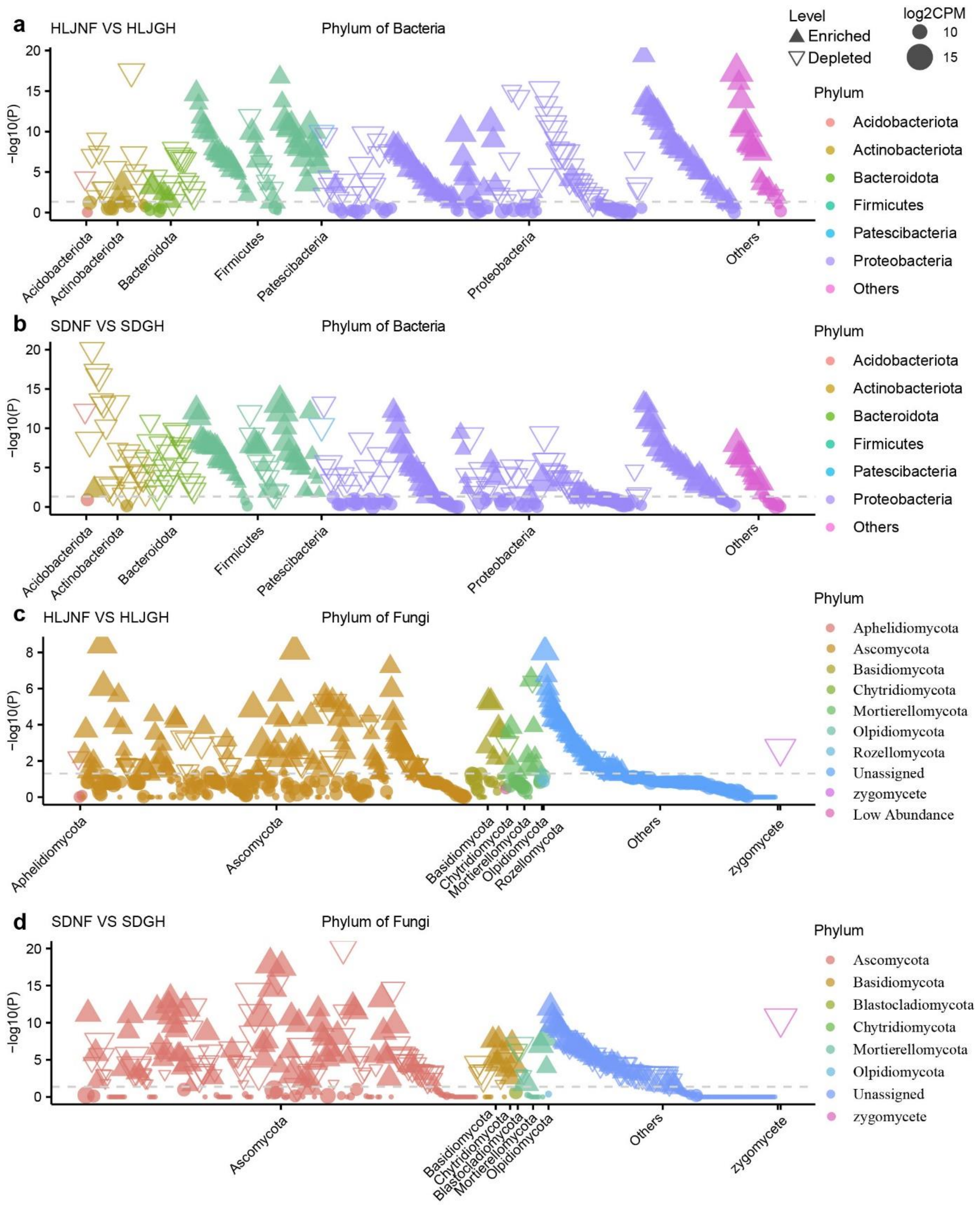

Supplementary Fig. 6 Manhattan plots presenting significantly enriched and depleted bacterial taxa in HLJNF tomato (**a**) and tomato SDNF (**b**) compared with those in HLJGH tomato and SDGH tomato respectively. Manhattan plots present significantly enriched and depleted fungal taxa in HLJNF tomato (**c**) and tomato SDNF (**d**) compared with those in HLJGH tomato and SDGH tomato respectively (FDR adjusted $P < 0.05$, two-sided Wilcoxon rank sum test).

274

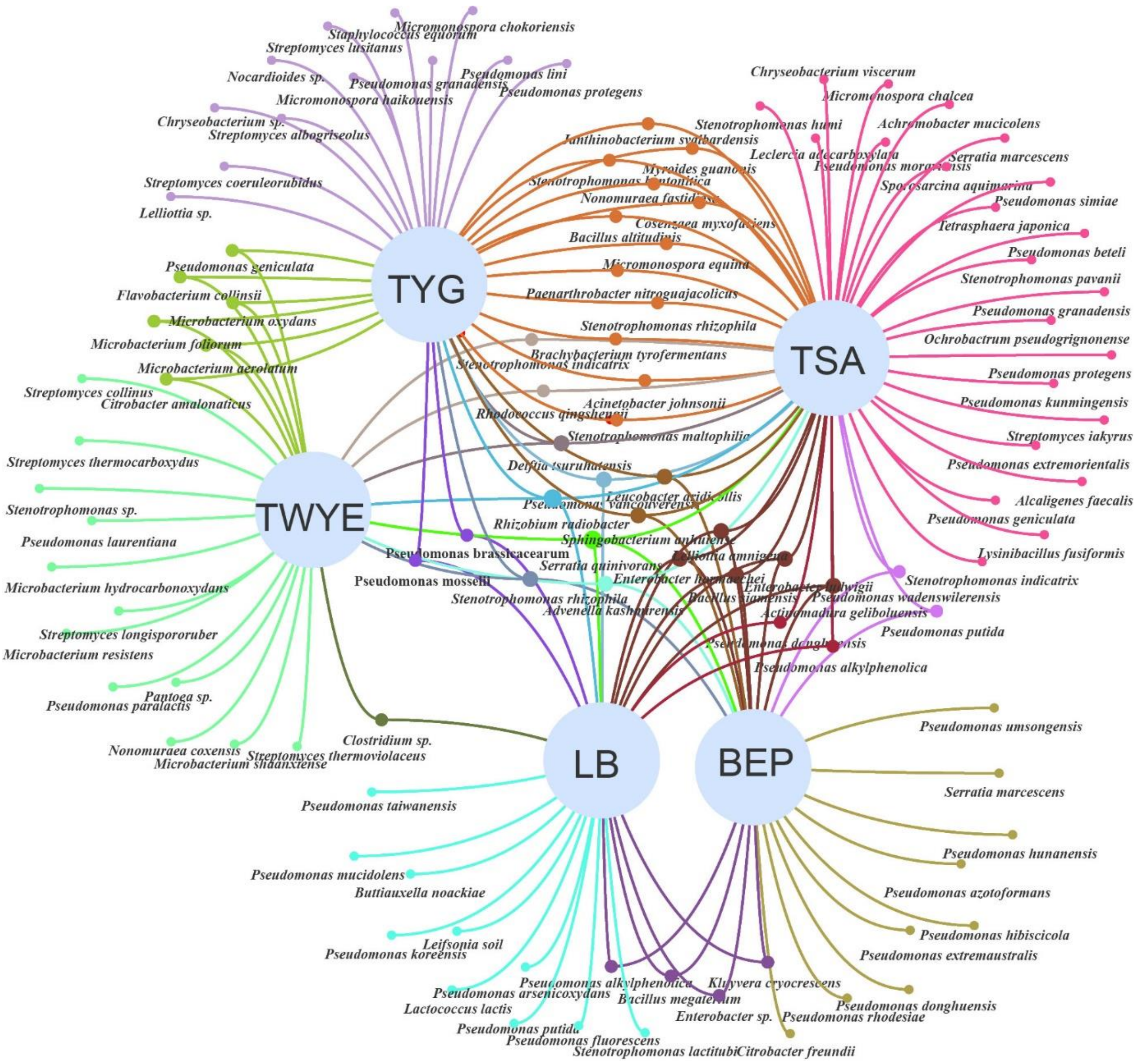

Supplementary Fig. 7 The Veen network plot presents shared and unique bacterial species isolated from five different culture media. BEP, Beef extract peptone; LB, Luria–Bertani; TSA, Tryptic Soy Agar; TWYE, Tap Water Yeast Extract; TYG, Tryptone Yeast extract Glucose Medium.

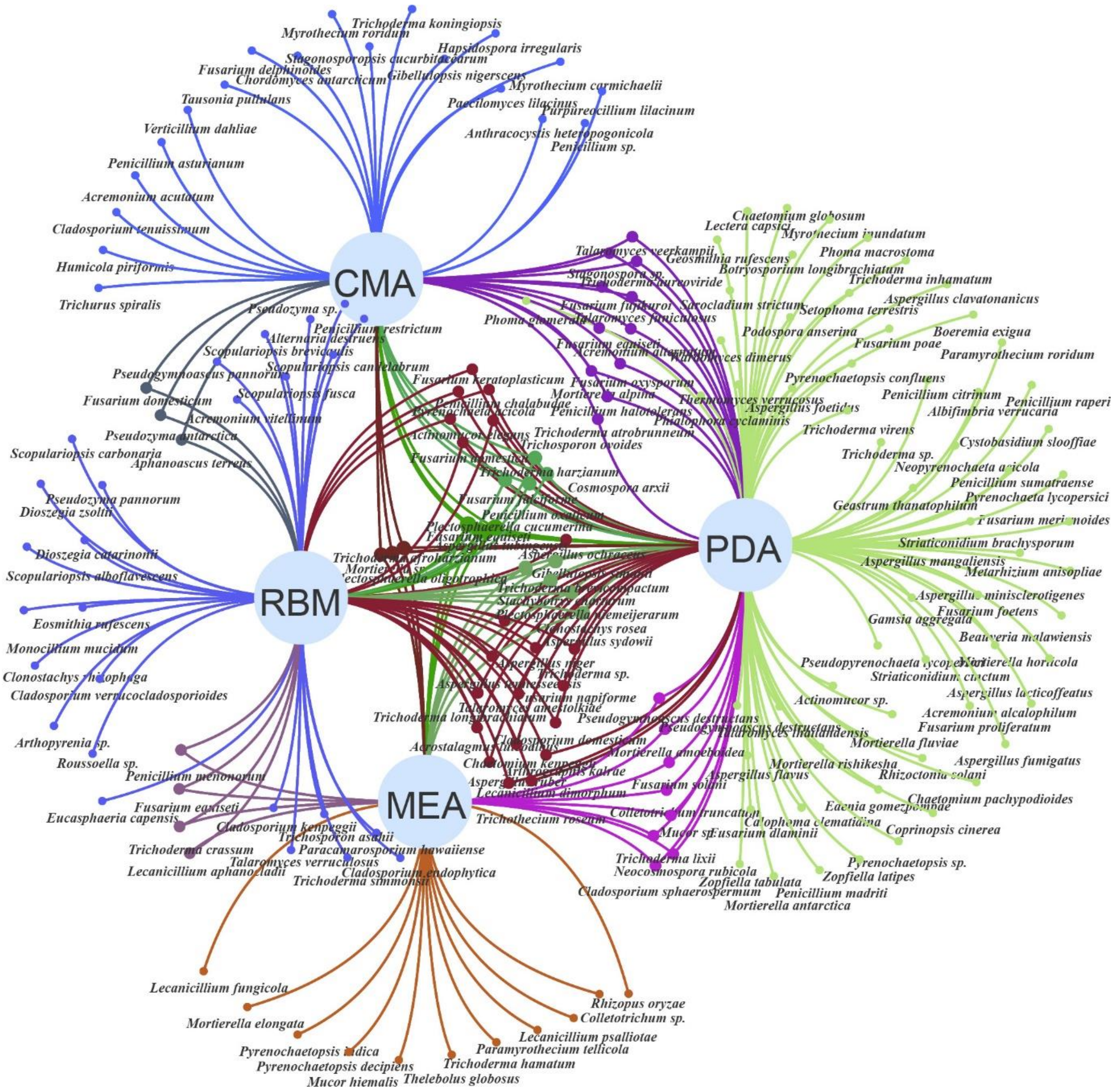

Supplementary Fig. 8 The Veen network plot presents shared and unique fungal species isolated from five different culture media. CMA, corn meal agar; PDA, Potato Dextrose Agar; RBM, Rose Bengal Medium; MEA, Malt Extract Agar.

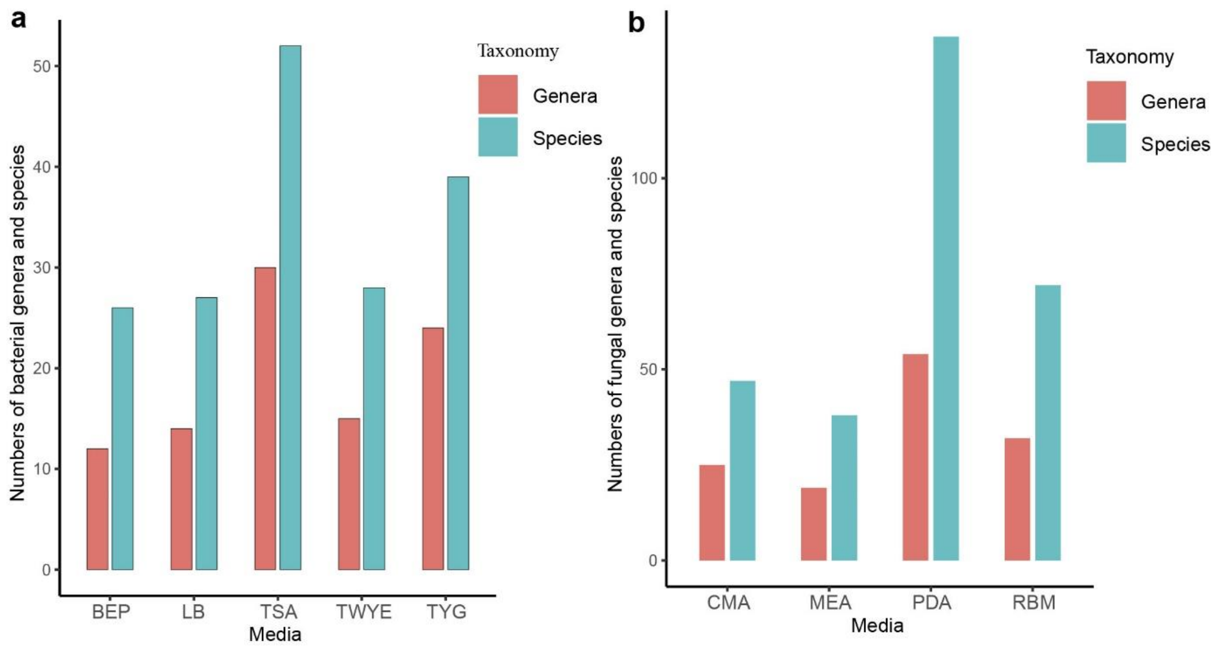

288

289 **Supplementary Fig. 9** The recovery of bacteria (**a**) and fungi (**b**) at species and genus levels
290 from different culture media were summarized.

291

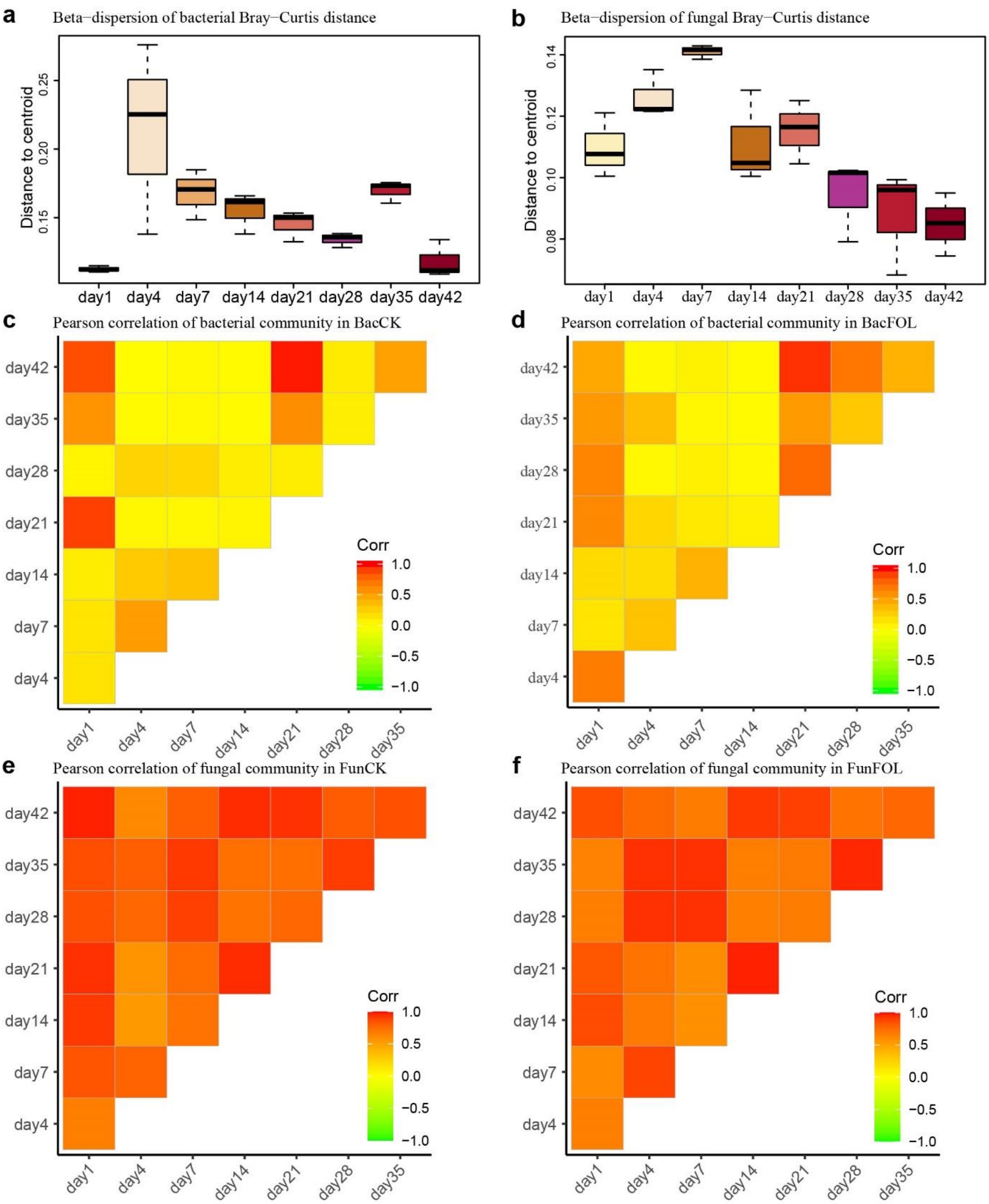

Supplementary Fig. 10 Beta dispersion (distance to group centroid) of bacterial communities (n =3 biologically independent plants) (**a**) and fungal communities (n =3 biologically independent plants) (**b**) among different time points of CrossKFOL SynComs. The central bars represent median values, tops and bottoms of boxes represent the 75th and 25th percentiles, and upper and lower whiskers extend to data no more than 1.5 times the interquartile range from the upper edge and lower edge of the box, respectively. Point value beyond this range is plotted as individual point. The pairwise correlations between different time points in BacCK

(**c**), BacFOL (**d**) SynComs of bacterial communities, and FunCK (**e**), FunFOL (**f**) SynComs of fungal communities were reflected by Pearson's correlation coefficients. In **c-f**, the yellow color indicates the value of Pearson's correlation coefficients lower than 0.5, and the red color indicates the value of Pearson's correlation coefficients greater than 0.5.

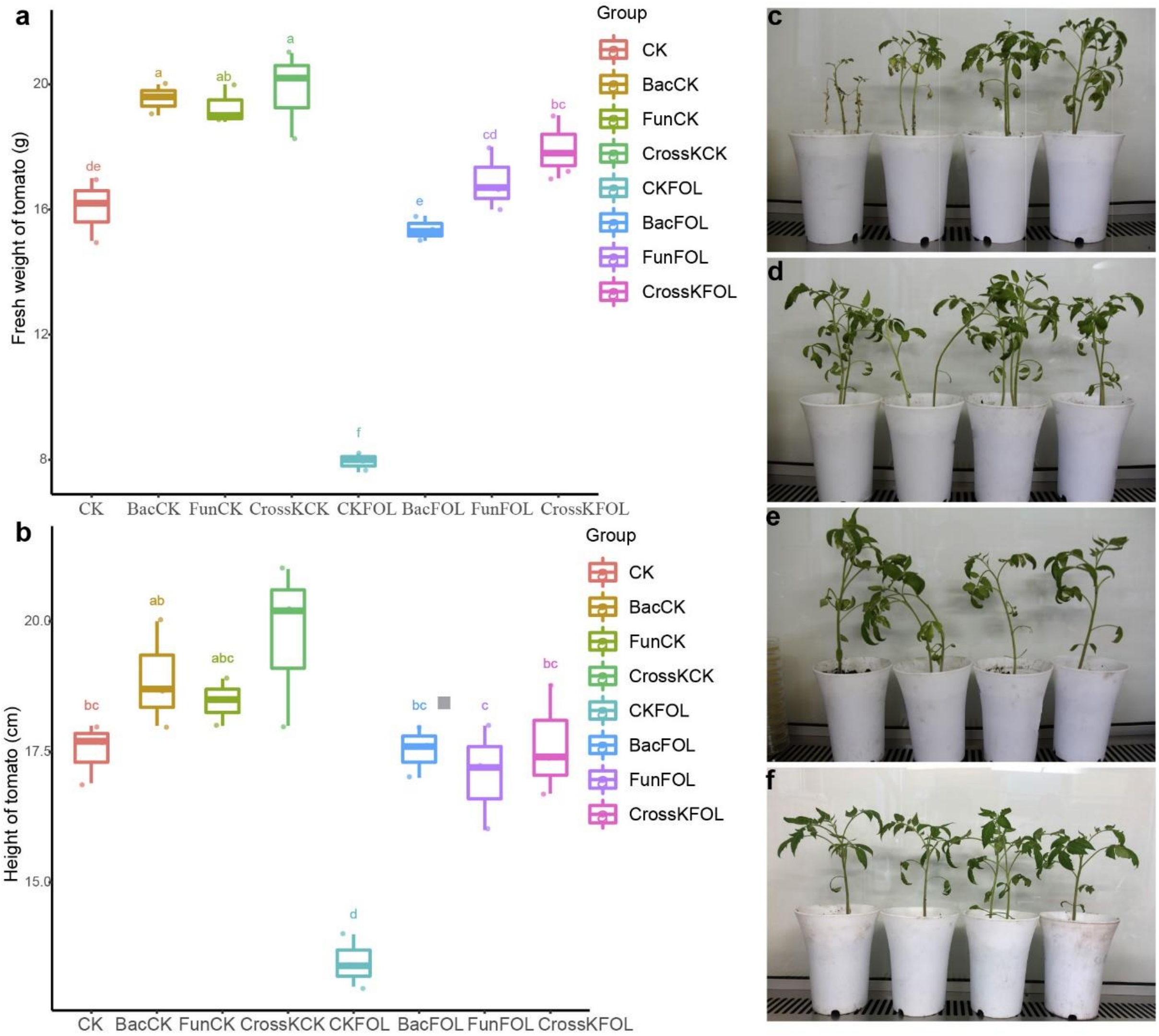

Supplementary Fig. 11 Fresh weight (**a**) and height (**b**)of tomato plants inoculated with CK SynComs, BacCK SynComs, FunCK SynComs, CrossKCK SynComs, CKFOL SynComs, BacFOL SynComs, FunFOL SynComs, CrossKFOL SynComs, and grem-free plants (CK) treatment at the day of 42 (P < 0.05, one-way ANOVA and Tukey HSD, n =3 biologically independent plants). Representative images of grem-free tomato seedlings inoculated only with *FOL* (**c**) *FOL* together with Bac SynComs (**d**), *FOL* together with Fun SynComs (**e**) and *FOL* together with CrossK (bacteria and fungi) SynComs (**f**). In g-h, the central bars represent median values, tops and bottoms of boxes represent the 75th and 25th percentiles, and upper and lower whiskers extend to data no more than 1.5 times of the interquartile range from the upper edge and lower edge of the box, respectively.

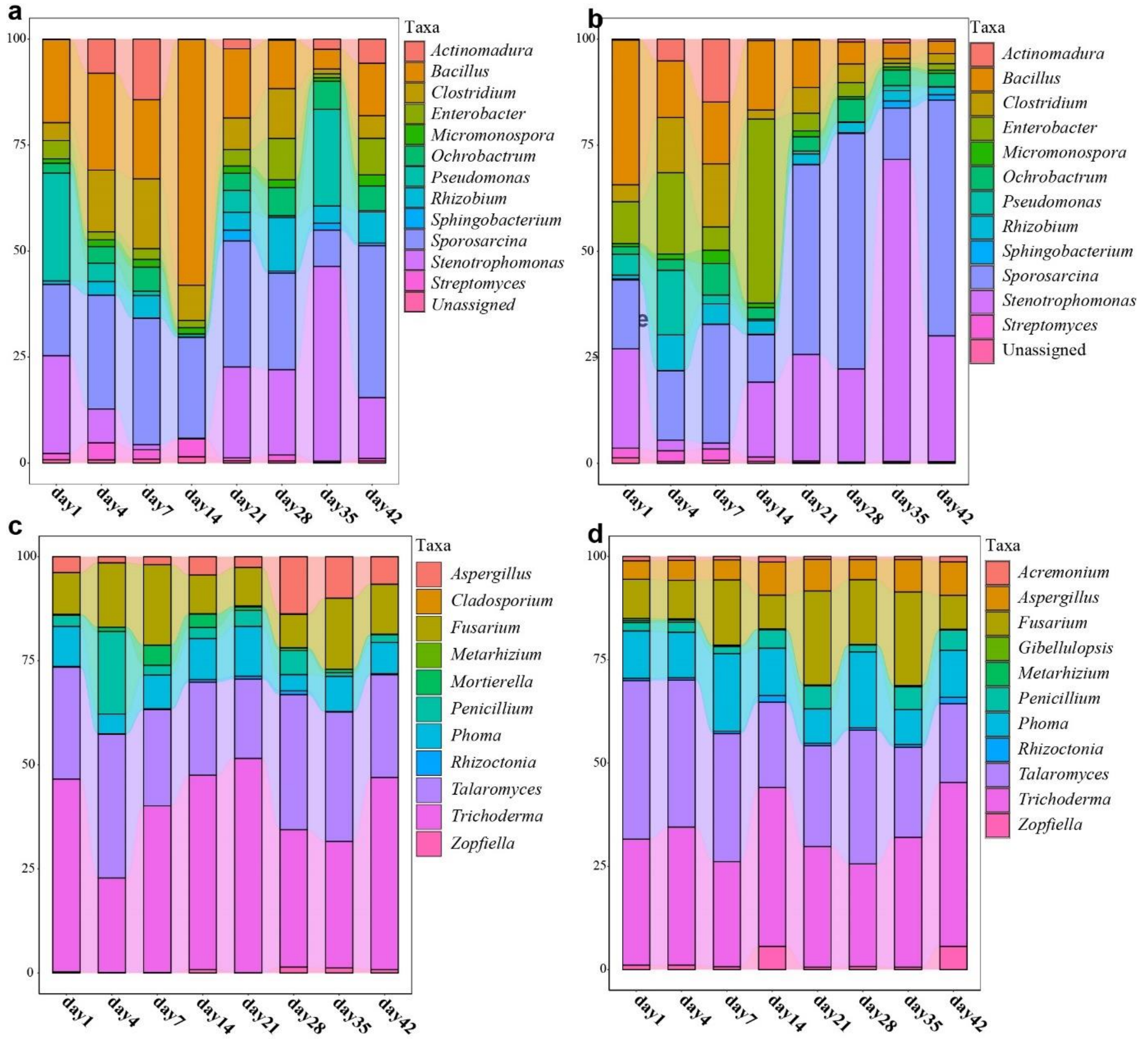

Supplementary Fig. 12 Bacterial abundance in Bac (**a**) and CrossK (**b**) SynComs, at the genus level, with the changes recorded at different growth time points. Fungal abundance in the Fun (**c**) and CrossK (**d**) SynComs, at the genus level, with the changes recorded at different growth time points.

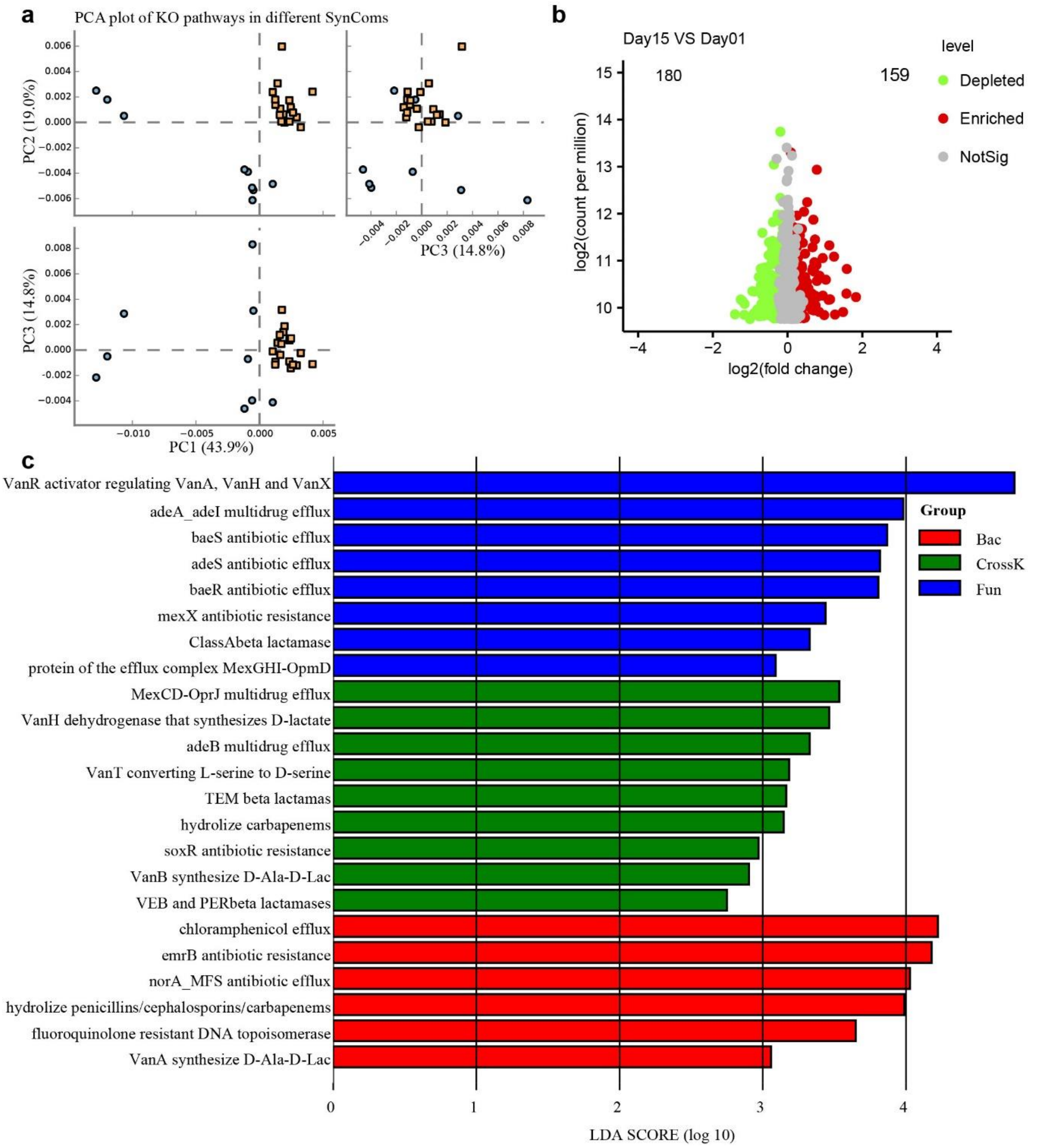

Supplementary Fig. 13 (**a**) PCA distance analysis of KO pathways in CrossK, Fun, and Bac SynComs inoculated with *FOL*, the PC1, PC2, and PC3, showed that the KO pathways of day 1 cluster separately from those on day 14. Yellow dots indicate tomato metagenomic samples of day 1 and blue dots indicate tomato metagenomic samples of day 14. (**b**) Volcano plots presenting significantly enriched and depleted KEGG pathways of Day 15 compared with those of Day 1 (FDR adjusted $P < 0.05$, two-sided Wilcoxon rank sum test). Red dots indicate enriched KEGG pathways of day 15, green dots indicate enriched KEGG pathways of day 1, and gray dots indicate non-significant KEGG pathways. (**c**) Indicator pathways with LDA

scores of 2 or greater in ResFam pathways associated with SynComs groups (red, Bac SynComs; green, CrossK SynComs; blue, Fun SynComs).
