# Supplementary material for: Cross-kingdom synthetic microbiota supports tomato suppression of Fusarium wilt disease
Source: Nat Commun. 2022 Dec 22;13:7890. doi: 10.1038/s41467-022-35452-6 (PMC9780251; doi:10.1038/s41467-022-35452-6)
Supplement: Supplementary file 23 — Reporting Summary [file 41467_2022_35452_MOESM23_ESM.pdf]

nature portfolio

Corresponding author(s): Lei Cai

Last updated by author(s): Nov 29, 2022

# Reporting Summary

Nature Portfolio wishes to improve the reproducibility of the work that we publish. This form provides structure for consistency and transparency in reporting. For further information on Nature Portfolio policies, see our Editorial Policies and the Editorial Policy Checklist.

## Statistics

For all statistical analyses, confirm that the following items are present in the figure legend, table legend, main text, or Methods section.

| n/a | Confirmed | |
|---|---|---|
| ☐ | ☒ | The exact sample size (*n*) for each experimental group/condition, given as a discrete number and unit of measurement |
| ☐ | ☒ | A statement on whether measurements were taken from distinct samples or whether the same sample was measured repeatedly |
| ☐ | ☒ | The statistical test(s) used AND whether they are one- or two-sided<br>*Only common tests should be described solely by name; describe more complex techniques in the Methods section.* |
| ☐ | ☒ | A description of all covariates tested |
| ☐ | ☒ | A description of any assumptions or corrections, such as tests of normality and adjustment for multiple comparisons |
| ☐ | ☒ | A full description of the statistical parameters including central tendency (e.g. means) or other basic estimates (e.g. regression coefficient) AND variation (e.g. standard deviation) or associated estimates of uncertainty (e.g. confidence intervals) |
| ☒ | ☐ | For null hypothesis testing, the test statistic (e.g. *F*, *t*, *r*) with confidence intervals, effect sizes, degrees of freedom and *P* value noted<br>*Give P values as exact values whenever suitable.* |
| ☒ | ☐ | For Bayesian analysis, information on the choice of priors and Markov chain Monte Carlo settings |
| ☒ | ☐ | For hierarchical and complex designs, identification of the appropriate level for tests and full reporting of outcomes |
| ☒ | ☐ | Estimates of effect sizes (e.g. Cohen's *d*, Pearson's *r*), indicating how they were calculated |

*Our web collection on statistics for biologists contains articles on many of the points above.*

## Software and code

Policy information about availability of computer code

| | |
|---|---|
| Data collection | 16S and ITS amplicon data was collected using an Illumina Miseq sequencer. RNA-Seq data and metagenomic was generated by sequencing on the Illumina Hiseq 4000 platform. |
| Data analysis | USEARCH v11.0<br>VSEARCH v2.8.1<br>R v4.0.0<br>NetShift (https://web.rniapps.net/netshift)<br>Bowtie2 v2.4.1<br>HUMAnN2 v2.8.1<br>MetaPhlAn2<br>Prokka<br>IQ-Tree<br>CD-HIT v4.8.1<br>eggnog-mapper v0.13.1<br>DIAMOND<br>STAMP<br>Linear discriminant analysis (LDA) effect size (LEfSe)<br>MultiQC v0.4<br>HISAT2 v2.2.0 |

SAMtools v1.3.1

For manuscripts utilizing custom algorithms or software that are central to the research but not yet described in published literature, software must be made available to editors and reviewers. We strongly encourage code deposition in a community repository (e.g. GitHub). See the Nature Portfolio guidelines for submitting code & software for further information.

## Data

Policy information about availability of data

All manuscripts must include a data availability statement. This statement should provide the following information, where applicable:

- Accession codes, unique identifiers, or web links for publicly available datasets
- A description of any restrictions on data availability
- For clinical datasets or third party data, please ensure that the statement adheres to our policy

All raw sequence data reported in this paper have been deposited in the Genome Sequence Read Archive in the National Genomics Data Center, China National Center for Bioinformation under GSA number: CRA006199. All the data could be viewed at https://ngdc.cncb.ac.cn/bioproject/browse/PRJCA008428 and downloaded through the weblink: https://bigd.big.ac.cn/gsa/browse/CRA006199.

## Human research participants

Policy information about studies involving human research participants and Sex and Gender in Research.

| | |
|---|---|
| Reporting on sex and gender | No Human research involved. |
| Population characteristics | No Human research involved. |
| Recruitment | No Human research involved. |
| Ethics oversight | No Human research involved. |

Note that full information on the approval of the study protocol must also be provided in the manuscript.

# Field-specific reporting

Please select the one below that is the best fit for your research. If you are not sure, read the appropriate sections before making your selection.

☒ Life sciences ☐ Behavioural & social sciences ☐ Ecological, evolutionary & environmental sciences

For a reference copy of the document with all sections, see nature.com/documents/nr-reporting-summary-flat.pdf

# Life sciences study design

All studies must disclose on these points even when the disclosure is negative.

| | |
|---|---|
| Sample size | Sample sizes were determined based on the Authors' experience of what is necessary to generate a convincing and compelling result. |
| Data exclusions | No data points were excluded from analysis in any experiment depicted in this manuscript. |
| Replication | The findings in this paper were remarkably reproducible. Every experiment was performed multiple times. |
| Randomization | Samples were randomly allocated into experimental groups. the samples of field, greenhouse and SynComs treatments were randomly collected. |
| Blinding | Individual plant constituting technical replicates was randomly numbered to be partially blind. When needed, some measurements, such as determination of disease index , were conducted independently by two group members to verify the results. |

# Reporting for specific materials, systems and methods

We require information from authors about some types of materials, experimental systems and methods used in many studies. Here, indicate whether each material, system or method listed is relevant to your study. If you are not sure if a list item applies to your research, read the appropriate section before selecting a response.

## Materials & experimental systems

| n/a | Involved in the study | |
|---|---|---|
| ☒ | ☐ | Antibodies |
| ☒ | ☐ | Eukaryotic cell lines |
| ☒ | ☐ | Palaeontology and archaeology |
| ☒ | ☐ | Animals and other organisms |
| ☒ | ☐ | Clinical data |
| ☒ | ☐ | Dual use research of concern |

## Methods

| n/a | Involved in the study | |
|---|---|---|
| ☒ | ☐ | ChIP-seq |
| ☒ | ☐ | Flow cytometry |
| ☒ | ☐ | MRI-based neuroimaging |
